# Systemic inflammatory biomarkers as predictive and prognostic factors in men with metastatic castration-refractory prostate cancer treated with docetaxel therapy: a comprehensive analysis in a German real-world cohort

**DOI:** 10.1007/s00432-022-04220-w

**Published:** 2022-08-08

**Authors:** Manuel Neuberger, Nora Goly, Janina Skladny, Veronica Milczynski, Christel Weiß, Frederik Wessels, Katja Nitschke, Britta Grüne, Caelán M. Haney, Friedrich Hartung, Jonas Herrmann, Jonas Jarczyk, Karl F. Kowalewski, Frank Waldbillig, Maximilian C. Kriegmair, Niklas Westhoff, Thomas S. Worst, Philipp Nuhn

**Affiliations:** 1 Department of Urology and Urologic Surgery, University Medical Centre Mannheim (UMM), Medical Faculty Mannheim, University of Heidelberg, Theodor-Kutzer-Ufer 1-3, 68167 Mannheim, Baden-Württemberg Germany; 2 Department of Medical Statistics and Biomathematics, Medical Faculty Mannheim, University of Heidelberg, Mannheim, Germany; 3 Department of Urology, University Hospital Leipzig, Leipzig, Germany

**Keywords:** Biomarker, Pre-treatment, Prognosis, Systemic inflammatory markers, Metastatic castration-refractory prostate cancer

## Abstract

**Purpose:**

Advances in therapy of metastatic castration-refractory prostate cancer (mCRPC) resulted in more therapeutic options and led to a higher need of predictive/prognostic biomarkers. Systemic inflammatory biomarkers could provide the basis for personalized treatment selection. This study aimed to assess the modified Glasgow Prognostic Score (mGPS), the neutrophile-to-lymphocyte ratio (NLR), the platelet-to-lymphocyte ratio (PLR) and the systemic immune-inflammation index (SII) in men with mCRPC under docetaxel.

**Methods:**

Patients with mCRPC and taxane chemotherapy at a tertiary care centre between 2010 and 2019 were screened retrospectively. The biomarkers mGPS, NLR, PLR and SII were assessed and analyzed for biochemical/radiologic response and survival.

**Results:**

We included 118 patients. Of these, 73 (61.9%) had received docetaxel as first-line, 31 (26.2%) as second-line and 14 (11.9%) as third-line treatment. For biochemical response, mGPS (odds ratio (OR) 0.54, *p* = 0.04) and PLR (OR 0.63, *p* = 0.04) were independent predictors in multivariable analysis. SII was significant in first-line cohort only (OR 0.29, *p* = 0.02). No inflammatory marker was predictive for radiologic response. In multivariable analysis, mGPS and NLR (hazard ratio (HR) 1.71 and 1.12, both *p* < 0.01) showed significant association with OS in total cohort and mGPS in the first-line cohort (HR 2.23, *p* < 0.01). Haemoglobin (Hb) and alkaline phosphatase (AP) showed several significant associations regarding 1 year, 3 year, OS and biochemical/radiologic response.

**Conclusions:**

Pre-treatment mGPS seems a promising prognostic biomarker. A combination of mGPS, NLR and further routine markers (e.g., Hb and AP) could yield optimized stratification for treatment selection. Further prospective and multicentric assessment is needed.

## Introduction

As the most common cancer in Europe and the second most common cancer among the male population worldwide, prostate cancer (PC) depicts a huge burden for the individual patient as well as for the health care system (Kreis et al. 2021; Michaeli and Michaeli 2022). While 74% of patients are diagnosed in a localized and curable stage, 13% present lymph node metastases and 7% already show distant metastases at time of diagnosis (Cancer Stat Facts 2021). Once metastasized, PC usually becomes resistant to luteinizing hormone-releasing antagonist or agonist therapy within 12 to 24 months leading to the stage of metastatic castration-resistant prostate cancer (mCRPC). In recent years, development and research has led to a broader field of treatment options in patients with mCRPC including docetaxel, abiraterone, enzalutamide, cabazitaxel, Olaparib, radium-223 and others (Cornford et al. 2021). With more therapeutic options available, the clinical decision-making process to choose the best treatment and the best sequence for the individual patient has become more difficult.

Docetaxel chemotherapy is a treatment that is recommended for fit patients only according to the EAU guidelines. For docetaxel chemotherapy, anaemia is the only laboratory prognostic biomarker for overall survival (OS), next to visceral metastases, pain, bone scan progression and prior estramustine (Armstrong et al. 2010). In addition to these clinical characteristics, the inflammatory response of patients has been described as predictor in cancer disease. To facilitate treatment decisions, different biomarkers have been developed and evaluated. These markers include a variety of routine laboratory markers, alterations in circulating cell-free DNA or genomic sequencing of tumor tissues (Stangl-Kremser et al. 2019; Neeb et al. 2021). However, the latter are cost- and time-expensive. The abundance of (inflammatory) biomarkers studied has led to the combination of biomarkers and combined prognostic scores like the modified Glasgow Prognostic Score (mGPS), the neutrophile-to-lymphocyte ratio (NLR), the platelet-to-lymphocyte ratio and the systemic immune-inflammation index (SII) as well in urological as in non-urological malignancies (Lee et al. 2015; Dolan et al. 2018; Wang et al. 2021). Recently, several studies provided evidence on the prognostic value of inflammatory markers in patients with mCRPC under various treatments including docetaxel (Donate-Moreno et al. 2020; Stangl-Kremser et al. 2020; Yamada et al. 2020).

This study’s aim was to investigate pre-treatment inflammatory biomarkers in a real-world cohort and add evidence on their suitability as predictors for treatment response and prognostic factors for survival in men with mCRPC receiving docetaxel chemotherapy in general and as first-line treatment.

## Materials and methods

### Study population and data collection

All patients who had received taxane-based chemotherapy at a tertiary university care centre (University Medical Centre Mannheim, Heidelberg University) in Germany between March 2010 and September 2019 were screened for docetaxel treatment in an mCRPC setting. All patients had continuous androgen-deprivation therapy. Laboratory routine markers were recorded for all cycles received at the centre. MGPS, NLR, PLR and SII were calculated as shown in Table 1.

**Table 1:** The modified Glasgow prognostic score (mGPS), neutrophile-to-lymphocyte ratio (NLR), platelet-to-lymphocyte ratio (PLR) and systemic immune-inflammation index (SII)

mGPS	
C-reactive protein ≤ 10 mg/l and any albumin value	0
C-reactive protein > 10 mg/l and albumin ≥ 35 g/l	1
C-reactive protein > 10 mg/l and albumin < 35 g/l	2
NLR	
$$\frac{\mathrm{neutrophil\,count }\,\left(\mathrm{reference\,range}: {4-10 \times 10}^{9}/\mathrm{L}\right)}{\mathrm{lymphocyte\,count\, }(\mathrm{reference\,range}: {1.1-3.2 \times 10}^{9}/\mathrm{L})}$$	
PLR	
$$\frac{\mathrm{platelet\,count }\,\left(\mathrm{reference\,range}: {150-400\mathrm{ x }10}^{9}/\mathrm{L}\right)}{\mathrm{lymphocyte\,count\, }(\mathrm{reference\,range}: {1.1-3.2\mathrm{ x }10}^{9}/\mathrm{L})}$$	
SII	
$$\frac{\mathrm{neutrophil\,count }\,\left(\mathrm{reference\,range}: {4-10\mathrm{ x }10}^{9}/\mathrm{L}\right)\times \mathrm{platelet\; count }\left(\mathrm{reference\,range}: {100-300\mathrm{ x }10}^{9}/\mathrm{L}\right)}{\mathrm{lymphocyte\,count\, }(\mathrm{reference\,range}: {1.1-3.2\mathrm{ x }10}^{9}/\mathrm{L})}$$	

Biochemical response was defined in two separate ways: PSA reduction of 30% and 50% comparing PSA value at initial administration and the value after the last received cycle or after cycle 6. Radiologic response to docetaxel was assessed by comparison of baseline staging and available imaging 4–6 weeks after last docetaxel application and was categorized in complete response (CR), partial response (PR), stable disease (SD) or progressive disease (PD). CR, PR and SD were analyzed individually and grouped. To assess survival status, death register query was carried out in April 2020, which marks the end date of survival analyses. Overall survival (OS), 1-year, 3-year and 5-year survival as well as radiologic response were assessed as binary parameters. Additionally, survival time or time to death was calculated. Demographic and clinical information were extracted from the medical records in the centre.

### Statistical analyses

Descriptive characteristics were performed for cohort characterization: for categorial variables, frequencies and proportions were determined, whereas medians and interquartile ranges (IQR) were computed for continuous variables. To assess the inflammatory markers as predictors for biochemical and radiologic response as well as survival Cochran-Armitage Trend Test was used. Furthermore, univariable logistic regression was used to test the impact of laboratory and clinical variables on the endpoints biochemical and radiologic response. Thereafter, multivariable logistic regression (backward selection) was used to evaluate for independent prognostic markers. For survival analysis, Kaplan–Meier analysis, log rank test and uni- and multivariable Cox-regression was conducted. All tests comparing two groups were two-sided. Statistical significance level was set at *α* = 0.05. Calculations were performed using the software SAS^®^(SAS Institute Inc., Cary, North Carolina, USA), release 9.4. For illustration GraphPad Prism9 (GraphPad Software, Inc, San Diego, California, USA) was used.

## Results

**Fig. 1:**
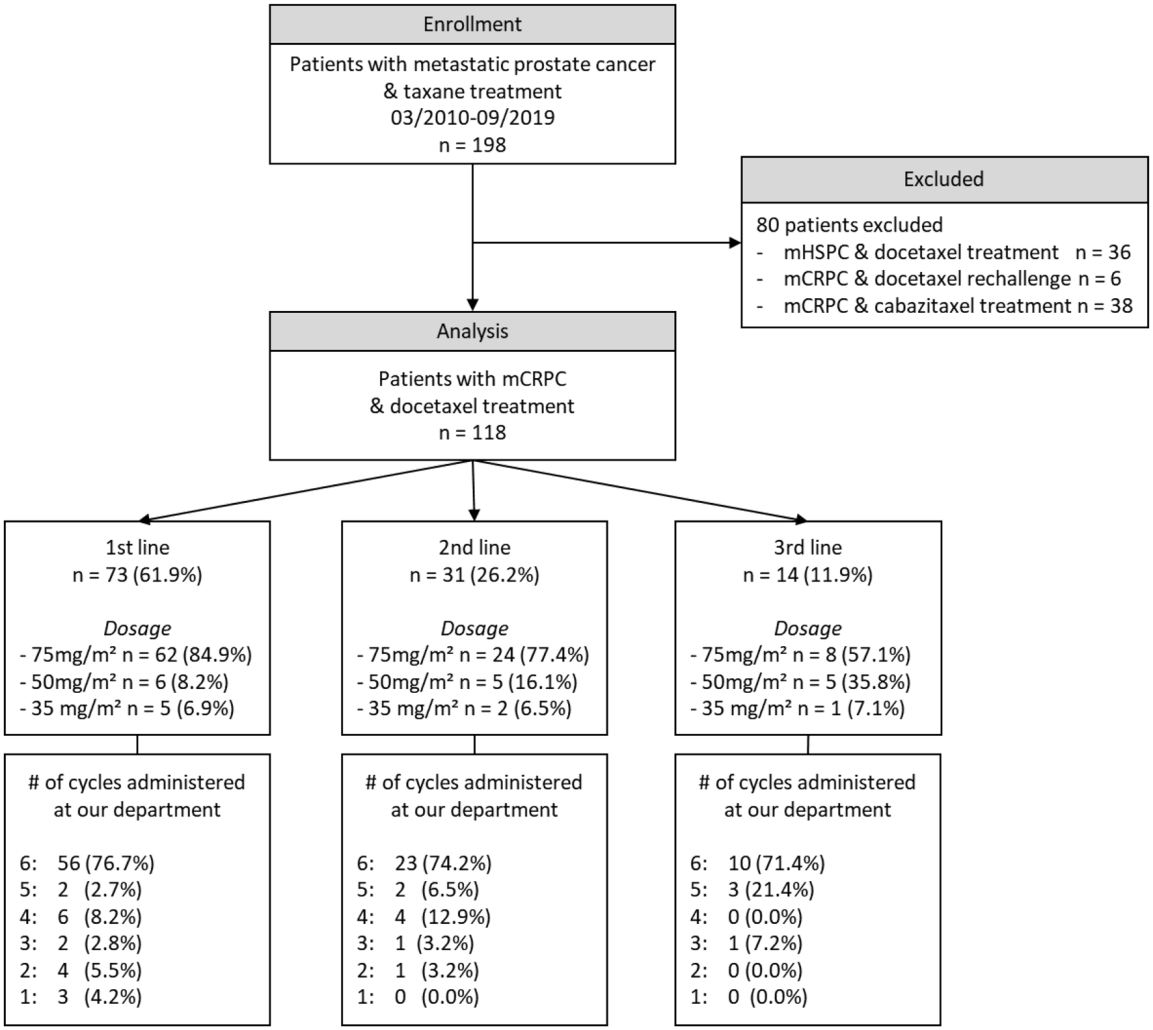
Flow-diagram and docetaxel treatment information of study cohort

A total of 118 patients were included in the analysis, of whom 73 (61.9%) received first-line docetaxel. A detailed clinical characterization of the cohort is shown in Fig. 1 and Table 2. Median survival was 18.5 (IQR 10.8–36.5) months in the total and 26.0 (IQR 12.0–49.5) months in first-line cohort.

**Table 2:** Baseline characteristics of study cohort

Characteristic	Cohort (*n* = 118)
Age [years], median [IQR]	72 [65–76]
Gleason score ≥ 8 (*n*, %)^a^	67 (69.8%)
PSA [ng/ml] at 1st cycle, median [IQR]^b^	82.0 [23.4–266.5]
Lymphatic metastases (*n*, %)	68 (57.6)
Osseous metastases (*n*, %)	101 (85.6)
Visceral metastases (*n*, %)	29 (24.6)
Hb [g/dl], median [IQR]^c^	12.2 [10.2–13.3]
mGPS^d^	
− 0 (*n*, %)	55 (57.9)
− 1 (*n*, %)	13 (13.7)
− 2 (*n*, %)	27 (28.4)
NLR, median [IQR]^e^	3.9 [2.74–5.82]
PLR, median [IQR]^e^	233.5 [141.5–312.4]
SII, median [IQR]^f^	160 [114.6–202.8]
AP [U/l], median [IQR]^g^	111.5 [74.8–230.0]
Albumin [g/dl], median [IQR]^e^	35.6 [31.8–38.7]
CRP [mg/l], median [IQR]^h^	15.4 [7.2–53.9]

^a^Data of 22 patient missing

^b^Data of 9 patients missing

^c^Data of 3 patients missing

^d^Data of 23 patients missing

^e^Data of 21 patients missing

^f^Data of 34 patients missing

^g^Data of 12 patients missing

^h^Data of 38 patients missing

### Total cohort

In the total cohort, mGPS (OR 0.54, 95% CI 0.30–0.98, *p* = 0.04) and PLR (OR 0.63, 95% CI 0.41–0.97, *p* = 0.04) remained independent predictors for biochemical response in multivariable logistic regression analysis. Regarding radiologic response, only Hb showed a significant association (OR 1.66, 95% CI 1.19–2.34, *p* < 0.01). As prognostic factors NLR, PLR and mGPS showed significant association with OS and 3-year survival in univariable Cox-regression analysis (all *p* < 0.01). However, of the four inflammatory markers examined, only mGPS showed significant association with OS (HR 1.71, 95% CI 1.25–2.36, *p* < 0.01) and 3-year survival (HR 1.63, 95% CI 1.17–2.27, *p* < 0.01) in multivariable Cox-regression analysis. Of notice, also Hb showed significant association with 3-year survival (0.77, 95% CI 0.66–0.91, *p* < 0.01) and remained the only significant prognostic factor for 1-year survival (0.54, 95% CI 0.43–0.68, *p* < 0.01) in multivariable analysis. Results are shown in Table 3.

**Table 3:** Uni- and multivariable logistic and Cox-regression in the total cohort (*n* = 118) to detect variables associated with **A** the biochemical response, **B** the radiologic response, **C** the overall survival (OS), **D** the 3-year survival and **E** the 1-year survival

**A**	Biochemical response (outcome: PSA reduction by 30%)					
Logistic regression:	Univariable			Multivariable^a^		
	HR	95% CI	*p*	OR	95% CI	*p*
Age (per year)	0.98	0.93–1.03	0.45			
Visceral disease (yes vs. no)	0.50	0.20–1.26	0.14			
Gleason Score ≥ 8 (yes vs. no)	1.09	0.45–2.68	0.85			
AP (per 100 units)	1.00	0.91–1.09	0.91			
Hb (per unit)	1.44	1.15–1.80	** < 0.01**			0.79
PSA (per 100 units)	0.89	0.78–1.01	0.08			0.28
NLR (per unit)	0.86	0.74–1.00	0.06			0.49
PLR (per 100 units)	0.61	0.43–0.87	** < 0.01**	0.63	0.41–0.97	**0.04**
SII (per 100 units)	0.63	0.32–1.23	0.17			
mGPS (per unit)	0.55	0.34–0.91	**0.02**	0.54	0.30–0.98	**0.04**

**B**	Radiologic Response (outcome: radiologic response)					
Logistic regression:	Univariable			Multivariable^a^		
	HR	95% CI	*p*	OR	95% CI	*p*
Age (per year)	1.00	0.94–1.07	0.85			
Visceral disease (yes vs. no)	0.82	0.24–2.77	0.75			
Gleason Score ≥ 8 (yes vs. no)	1.01	0.31–3.28	0.98			
AP (per 100 units)	0.84	0.61–1.15	0.27			
Hb (per unit)	1.66	1.19–2.34	** < 0.01**	1.66	1.19–2.34	** < 0.01**
PSA (per 100 units)	0.93	0.78–1.11	0.41			
NLR (per unit)	0.79	0.60–1.03	0.08			0.38
PLR (per 100 units)	0.71	0.48–1.11	0.13			
SII (per 100 units)	0.66	0.25–1.77	0.41			
mGPS (per unit)	1.00	0.56–1.80	1.00			

**C**	Overall Survival (outcome: death)					
Cox-regression:	Univariable			Multivariable^a^		
	HR	95% CI	*p*	HR	95% CI	*p*
Age (per year)	1.01	0.98–1.04	0.67			
Visceral disease (yes vs. no)	1.67	1.04–2.66	**0.03**			0.24
Gleason Score ≥ 8 (yes vs. no)	0.94	0.57–1.57	0.82			
AP (per 100 units)	1.05	1.02–1.08	** < 0.01**			0.14
Hb (per unit)	0.77	0.68–0.86	** < 0.01**			0.44
PSA (per 100 units)	1.08	1.03–1.12	** < 0.01**			0.11
NLR (per unit)	1.10	1.02–1.17	**0.01**	1.12	1.03–1.22	** < 0.01**
PLR (per 100 units)	1.25	1.07–1.45	** < 0.01**			0.50
SII (per 100 units)	1.20	0.84–1.72	0.32			
mGPS (per unit)	1.82	1.39–2.39	** < 0.01**	1.71	1.25–2.36	** < 0.01**

**D**	3-year survival (outcome: death after 3 years)					
Cox-regression:	Univariable			Multivariable^a^		
	HR	95% CI	*p*	HR	95% CI	*p*
Age (per year)	1.00	0.97–1.03	0.92			
Visceral disease (yes vs. no)	2.14	1.32–3.47	** < 0.01**			0.29
Gleason Score ≥ 8 (yes vs. no)	1.03	0.59–1.79	0.92			
AP (per 100 units)	1.05	1.02–1.08	** < 0.01**			0.29
Hb (per unit)	0.73	0.65–0.83	** < 0.01**	0.77	0.66–0.91	** < 0.01**
PSA (per 100 units)	1.08	1.03–1.12	** < 0.01**			0.25
NLR (per unit)	1.11	1.03–1.12	** < 0.01**			0.05
PLR (per 100 units)	1.28	1.01–1.50	** < 0.01**			0.51
SII (per 100 units)	1.11	0.75–1.65	0.60			
mGPS (per unit)	2.05	1.15–2.75	** < 0.01**	1.63	1.17–2.27	** < 0.01**

**E**	1-year survival (outcome: death after 1 year)					
Cox-regression:	Univariable			Multivariable^a^		
	HR	95% CI	*p*	HR	95% CI	*p*
Age (per year)	0.98	0.94–1.03	0.45			
Visceral disease (yes vs. no)	3.26	1.69–6.30	** < 0.01**			0.28
Gleason Score ≥ 8 (yes vs. no)	2.23	0.85–5.87	0.10			
AP (per 100 units)	1.06	1.01–1.10	**0.01**			0.38
Hb (per unit)	0.57	0.47–0.69	** < 0.01**	0.54	0.43–0.68	** < 0.01**
PSA (per 100 units)	1.08	1.03–1.13	** < 0.01**			0.21
NLR (per unit)	1.11	1.02–1.22	**0.02**			0.20
PLR (per 100 units)	1.44	1.21–1.71	** < 0.01**			0.08
SII (per 100 units)	1.55	0.91–2.66	0.11			
mGPS (per unit)	3.85	2.30–6.44	** < 0.01**			0.59

*HR* hazard ratio, *OR* odds ratio, *CI* confidence interval

^a^Backward selection

### Subgroup of patients with docetaxel as first-line therapy

In the subgroup that received first-line docetaxel, SII (*p* = 0.05) and PLR (*p* = 0.02) showed significant association with biochemical response. SII remained the only independent and significant predictor in multivariable logistic regression analysis (OR 0.29, 95% CI 0.10.-0.82, *p* = 0.02). Matching the results from the total cohort, none of the four examined inflammatory markers but only Hb (OR 1.48, 95% CI 1.01–2.14, *p* = 0.04) showed significant prediction for radiologic response. As prognostic factor for survival, mGPS as the only one of the four inflammatory markers showed significant association in univariable Cox-regression with OS, 3-year and 1-year survival (all *p* < 0.01) and remained an independent prognostic marker in multivariable Cox-regression analysis for OS (HR 2.24, 95% CI 1.50–3.36, *p* < 0.01), 3 year (HR 2.74, 95% CI 1.74–4.37, *p* < 0.01) and 1-year survival (HR 5.24, 95% CI 2.39–11.51, *p* < 0.01). Of the other variables studied, AP (*p* = 0.02) and Hb (*p* < 0.01) showed a significant association with OS, but AP only remained significant in multivariable analysis (*p* = 0.01). Furthermore, for 3-year survival, visceral disease (*p* < 0.01), AP (0.02) and Hb (< 0.01) showed significant association as did visceral disease and Hb (both *p* < 0.01) for 1-year survival prediction. In multivariable Cox-regression analysis, only AP remained an independent prognostic factor for 3-year survival (HR 1.07 (per 100 units), 95% CI 1.02–1.12, *p* < 0.01). Detailed results are shown in Appendix 1.

### Survival analysis

**Fig. 2:**
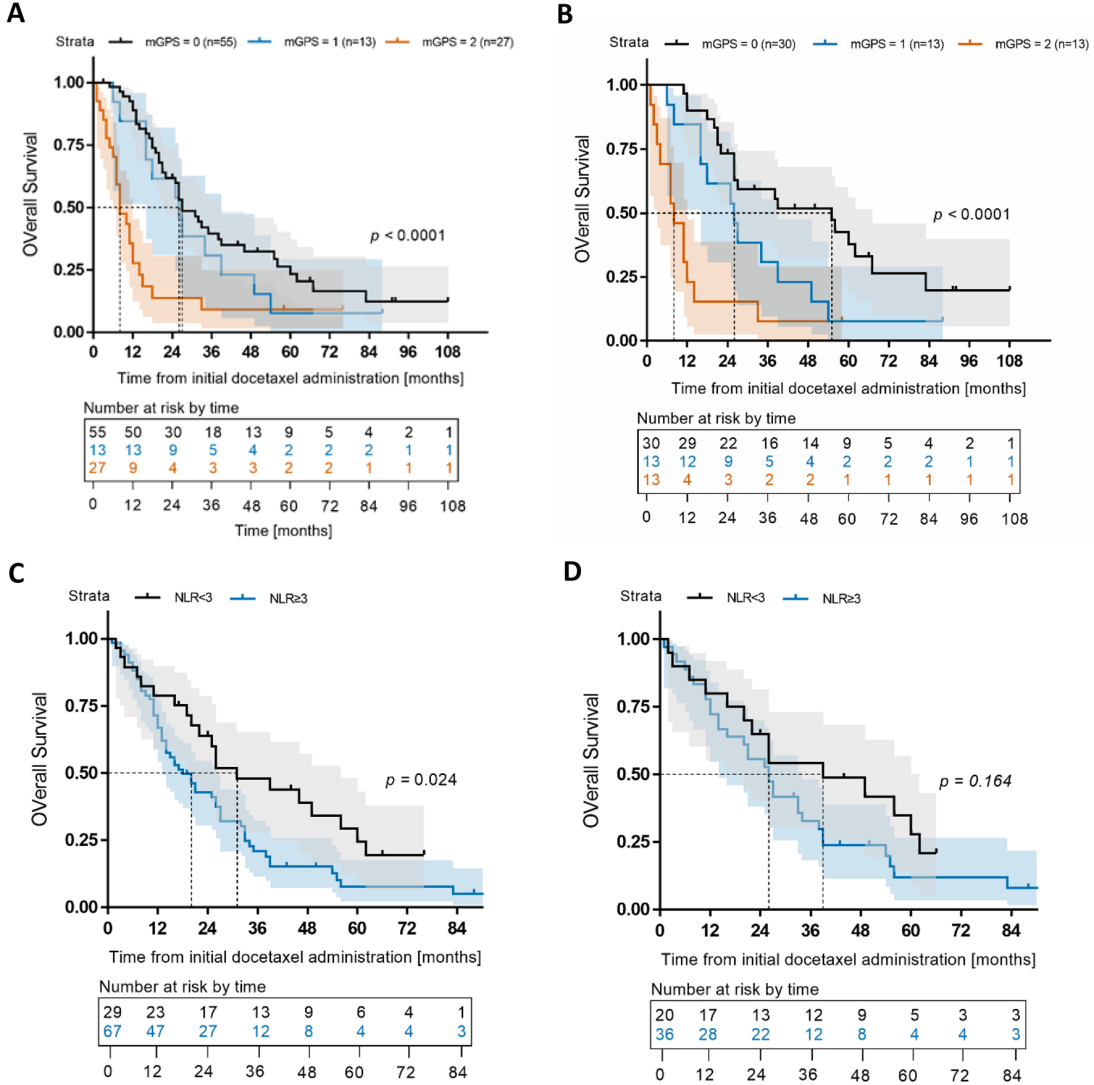
Kaplan–Meier analysis of overall survival (OS) [months] depending on **A** mGPS in the total cohort **B** mGPS in the docetaxel first-line subgroup **C** NLR in the total cohort and **D** NLR in the docetaxel first-line subgroup

Kaplan–Meier analysis revealed longer OS in patients with lower mGPS than those with higher mGPS in the total cohort (median survival: mGPS 0 = 27 months, mGPS 1 = 26 months, mGPS 2 = 8 months, *p* < 0.01) and in the subgroup of patients with first-line docetaxel treatment (median survival: mGPS 0 = 55 months, mGPS 1 = 26 months, mGPS 2 = 8 months, *p* < 0.01). Using the commonly accepted NLR cut-off of 3, Kaplan–Meier analysis of the total cohort additionally showed a significantly poorer survival for patients with an NLR ≥ 3 (median survival 18 vs. 31 months, HR 1.74, 95% CI 1.08–2.82, *p* = 0.02). In the subgroup of patients receiving docetaxel as first-line treatment, the NLR cut-off of 3 did not reach significance (median survival 26 vs. 39 months, HR 1.54, 95% CI 0.84–2.88, *p* = 0.16). Results are shown in Fig. 2.

## Discussion

The recent advances in therapy of mCRPC with more therapeutic options becoming available have led to a higher need of predictive and prognostic biomarkers for personalized treatment selection. Inflammation is one of cancers’ hallmarks and systemic inflammatory biomarkers represent the body’s reaction to disease. Furthermore, they are easily available and inexpensive. Even though there has been more evidence on inflammatory biomarkers in mCRPC lately (Stangl-Kremser et al. 2020; Yamada et al. 2020), the available guidelines on PC (e.g., EAU, AUA, German S3) do not contain any statement in this regard and recommend Hb, AP and LDH as baseline laboratory biomarkers only (Cornford et al. 2021; Leitlinienprogramm Onkologie 2021; Lowrance et al. 2021). In this study, we aimed to provide further evidence for systemic inflammatory markers as predictive and prognostic factors.

Regarding biochemical response, the results yielded in this study do not coincide between the total cohort and the subgroup with docetaxel as first-line therapy: whereas PLR and mGPS remained independent predictors for biochemical response multivariable analysis in the total cohort, SII was the only predictor in the cohort that received docetaxel as first-line treatment. As predictive biomarkers for radiologic response, none of the inflammatory markers but only Hb showed an independent predictive value as assessed in multivariable analysis. Regarding survival, the results where conclusive: mGPS remained the only independent prognostic factor in both cohorts for OS and 3-year survival. In the subgroup treated with docetaxel as first-line treatment, mGPS additionally showed significant association with 1-year survival. NLR and PLR showed significant results as prognostic factors regarding 1 year, 3 year, and OS in uni- but not in multivariable analyses in the total cohort but not the first-line subgroup. Survival analysis revealed a longer survival for patients with lower mGPS in both groups as well as for patients with an NLR > 3 in the total cohort. SII did not reach significance as a prognostic marker in any analysis. Of the non-inflammatory markers/variables studied, AP and Hb were identified as independent prognostic markers for survival, which matches existing evidence. Taken together, our results particularly underline the additional value of mGPS and NLR as pragmatic prognostic biomarkers next to Hb.

In a prospective cohort-study including 80 patients, Donate-Moreno et al. in 2020 investigated inflammatory markers in mCRPC under various treatment and could show a negative correlation of NLR, PLR and SII with survival time (Donate-Moreno et al. 2020). Yamada et al. in 2020 retrospectively analyzed 196 patients with mCRPC from multiple institutions and built an inflammation index based on derived neutrophiles/(leukocytes minus neutrophils) ratio (dNLR) and LDH. They could show that stratification by their inflammation index led to longer OS in the “Good inflammatory index” group (Yamada et al. 2020). In their 2019 meta-analysis on pretreatment systemic inflammatory markers, Peng et al. included 32 studies. Sub-analysis of the 13 studies investigating patients with mCRPC and undergoing chemotherapy revealed NLR as possible effective predictive biomarker (Peng and Luo 2019). Fan et al. could show that a high SII remained a significant predictor of OS, radiologic and biochemical progression free survival in their 2018 publication including 104 patients that had received either abiraterone followed by docetaxel or vice versa (Fan et al. 2018). The mGPS has been shown to be correlated with OS (Linton et al. 2013, Ando et al. 2021), progression of mCRPC (Stangl-Kremser et al. 2020) and poorer relative survival independent of age as well as 5-year survival (Shafique et al. 2012). For patients with metastatic hormone-sensitive PC, mGPS could be shown as a predictive and prognostic biomarker for radiologic response and OS (Neuberger et al. 2022). In metastatic penile cancer, the mGPS was associated significantly with treatment response (Draeger et al. 2021) and in pretreated advanced urinary tract cancer the combination of SII, programme death-ligand 1(PD-L1) and LDH showed itself useful as a prognostic tool (Fornarini et al. 2021).

Next to urological cancers, systemic inflammatory markers have been evaluated in multiple other malignancies: For example, a recent meta-analysis showed high levels of NLR, GPS and CRP to be associated with worsened prognosis in patients with osteosarcoma (Song et al. 2021). Another systematic review and meta-analysis showed, that among others, mGPS, NLR, PLR, SII have a moderate predictive ability in OS, disease-free survival and cancer-specific survival in oesophageal cancer (Jiang et al. 2021). In colorectal cancer, NLR could be confirmed as prognostic biomarker for OS (Naszai et al. 2021) and in gastric cancer poor survival was associated with CRP, NLR and GPS/mGPS (Kim et al. 2020) in other systematic reviews and meta-analyses.

Furthermore, the results of this study add to the existing evidence for the predictive and prognostic value of systemic inflammatory markers in mCRPC. From the four markers that we examined, mGPS seems to be the most promising one as it allows a stratification and yielded significant results associated with patient survival as well in the total cohort as in the subgroup of patients with docetaxel as first-line therapy. Additionally, mGPS combines an inflammatory component (CRP) with a surrogate nutritional assessment (albumin). A recent study showed that the combination of the body mass index and albumin in patients with mCRPC treated with abiraterone is predictive of OS (Pan et al. 2021) regardless of previous chemotherapies. This underlines the importance of the assessment and intervention of nutritional status in terms of supportive therapy in this patient group. Other studies could show that low albumin levels correlate with poor prognosis in metastatic renal cell carcinoma (Zhou et al. 2022). Next to mGPS, NLR shows promising results regarding prediction of survival in patients with mCRPC, which has been shown in various studies (Peng and Luo 2019). Considering Hb, which also showed a significant association with patients’ survival, a combined score of mGPS, NLR and Hb could be a promising tool for survival prediction and thereby help to assess the patient for a more personalized treatment selection.

## Limitations

The retrospective design of the study and the fact that it is monocentric limit the significance of this study. Furthermore, the rather small sample size and the fact that one of the necessary variables for mGPS was missing in 23 patients limits the statistical power and generalisability. Additionally, there was no assessment of other diseases (e.g., secondary malignancies, infections, bleeding) as only disease-specific characteristics were collected. Of note, the performed death register query did not assess the reason of death, which means, that patients could have died from PC unspecific causes. Furthermore, the cohort is heterogeneous in terms of received cancer therapies: not only has docetaxel been given as first-, second- or third-line therapy, but also the numbers of administered cycles as well as the difference in existence and numbers of previous and following PC therapies differ among these patients. Considering these limitations, this could also strengthen our findings and make them more robust and pragmatic.

Another limitation is the fact that the imaging was not evaluated according to Response Evaluation Criteria In Solid Tumors (RECIST).

## Conclusion

This study evaluated mGPS, NLR, PLR and SII as predictive and prognostic biomarkers in patients with mCRPC who receive docetaxel. Pre-treatment mGPS seems the most promising independent and pragmatic biomarker regarding survival prediction. A combination of mGPS, NLR and Hb could yield an optimized stratification. The potential of mGPS, PLR, and SII as predictors for biochemical response remains unclear. Further assessment in prospective and multicentric studies is needed.

## Data availability statement

Data are available for bona fide researchers who request it from the authors.

## Appendix 1

Uni- and multivariable logistic and Cox-regression in the first-line docetaxel subgroup (*n* = 73) to detect variables associated with **A** the biochemical response **B** the radiologic response **C** the overall survival (OS) **D** the 3-year survival and **D** the 1-year survival

**Table:**

**A**	Biochemical response (outcome: PSA reduction by 30%)					
Logistic regression:	Univariable			Multivariable^a^		
	HR	95% CI	*p*	OR	95% CI	*p*
Age (per year)	0.98	0.92–1.06	0.67			
Visceral disease (yes vs. no)	0.50	0.16–1.65	0.26			
Gleason Score ≥ 8 (yes vs. no)	0.77	0.22–2.69	0.68			
AP (per 100 units)	1.04	0.90–1.20	0.60			
Hb (per unit)	1.36	1.03–1.80	**0.03**			0.13
PSA (per 100 units)	0.92	0.74–1.15	0.48			
NLR (per unit)	0.93	0.77–1.13	0.47			
PLR (per 100 units)	0.62	0.38–1.00	**0.05**			0.83
SII (per 100 units)	0.29	0.10–0.82	**0.02**	0.29	0.10–0.82	**0.02**
mGPS (per unit)	0.53	0.26–1.10	0.09			0.56

**B**	Radiologic response (outcome: radiologic response)					
logistic regression:	Univariable			Multivariable^a^		
	HR	95% CI	*p*	OR	95% CI	*p*
Age (per year)	1.00	0.92–1.09	0.97			
Visceral disease (yes vs. no)	1.09	0.25–4.86	0.91			
Gleason Score ≥ 8 (yes vs. no)	0.85	0.18–4.01	0.84			
AP (per 100 units)	0.92	0.71–1.18	0.51			
Hb (per unit)	1.48	1.01–2.14	**0.04**	1.48	1.01–2.14	**0.04**
PSA (per 100 units)	0.98	0.74–1.28	0.87			
NLR (per unit)	0.91	0.69–1.19	0.47			
PLR (per 100 units)	0.96	0.53–1.74	0.88			
SII (per 100 units)	0.71	0.19–2.64	0.60			
mGPS (per unit)	0.97	0.40–2.35	0.95			

**C**	Overall Survival (outcome: death)					
Cox-regression:	Univariable			Multivariable^a^		
	HR	95% CI	*p*	HR	95% CI	*p*
Age (per year)	1.01	0.97–1.05	0.68			
Visceral disease (yes vs. no)	1.73	0.96–3.14	0.07			0.31
Gleason Score ≥ 8 (yes vs. no)	1.04	0.52–2.07	0.92			
AP (per 100 units)	1.05	1.00–1.09	**0.02**	1.06	1.01–1.11	**0.01**
Hb (per unit)	0.80	0.70–0.92	** < 0.01**			0.44
PSA (per 100 units)	1.08	0.98–1.18	0.14			
NLR (per unit)	1.06	0.96–1.12	0.23			
PLR (per 100 units)	1.08	0.85–1.36	0.55			
SII (per 100 units)	1.13	0.72–1.77	0.60			
mGPS (per unit)	2.30	1.57–3.37	** < 0.01**	2.24	1.50–3.36	** < 0.01**

**D**	3-year survival (outcome: death after 3 years)					
Cox-regression:	Univariable			Multivariable^a^		
	HR	95% CI	*p*	HR	95% CI	*p*
Age (per year)	1.00	0.96–1.04	0.99			
Visceral disease (yes vs. no)	2.36	1.26–4.43	** < 0.01**			0.83
Gleason Score ≥ 8 (yes vs. no)	1.30	0.56–3.05	0.54			
AP (per 100 units)	1.05	1.01–1.09	**0.02**	1.07	1.02–1.12	** < 0.01**
Hb (per unit)	0.77	0.66–0.90	** < 0.01**			0.61
PSA (per 100 units)	1.05	0.94–1.18	0.38			
NLR (per unit)	1.08	0.97–1.19	0.17			
PLR (per 100 units)	1.09	0.84–1.43	0.52			
SII (per 100 units)	0.96	0.57–1.63	0.89			
mGPS (per unit)	2.65	1.73–4.06	** < 0.01**	2.74	1.74–4.37	** < 0.01**

**E**	1-year survival (outcome: death after 1 year)					
Cox-regression:	Univariable			Multivariable^a^		
	HR	95% CI	*p*	HR	95% CI	*p*
Age (per year)	1.01	0.95–1.08	0.66			
Visceral disease (yes vs. no)	3.39	1.37–8.37	** < 0.01**			0.90
Gleason Score ≥ 8 (yes vs. no)	2.30	0.51–10.3	0.28			
AP (per 100 units)	1.04	0.98–1.10	0.19			
Hb (per unit)	0.68	0.53–0.86	** < 0.01**			0.37
PSA (per 100 units)	1.10	0.95–1.29	0.21			
NLR (per unit)	1.08	0.93–1.26	0.32			
PLR (per 100 units)	1.38	0.94–2.04	0.10			
SII (per 100 units)	1.38	0.64–2.96	0.41			
mGPS (per unit)	5.24	2.39–11.51	** < 0.01**	5.24	2.39–11.51	** < 0.01**

*HR* hazard ratio, *OR* odds ratio, *CI* confidence interval

^a^backward selection
